# Prevalence and molecular characterisation of human adenovirus in diarrhoeic children in Tanzania; a case control study

**DOI:** 10.1186/s12879-014-0666-1

**Published:** 2014-12-12

**Authors:** Sabrina John Moyo, Kurt Hanevik, Bjørn Blomberg, Oyvind Kommedal, Svein Arne Nordbø, Samuel Maselle, Nina Langeland

**Affiliations:** Department of Clinical Science, University of Bergen, Bergen, Norway; Department of Microbiology and Immunology, Muhimbili University of Health and Allied Sciences, Dar es Salaam, Tanzania; Department of Microbiology, Haukeland University Hospital, Bergen, Norway; National Centre for Tropical Infectious Diseases, Haukeland University Hospital, Bergen, Norway; Department of Medical Microbiology, University Hospital of Trondheim, Trondheim, Norway; Institute of Laboratory Medicine, Children’s and Women’s Health, Norwegian University of Science and Technology, Trondheim, Norway; Department of Medicine, Haukeland University Hospital, Bergen, Norway

**Keywords:** Adenovirus, PCR, HIV, Seasonality, Tanzania

## Abstract

**Background:**

Human adenovirus (HAdV) causes acute diarrhoea sporadically, as well as in outbreaks. Understanding the prevalence and types of HAdV in diarrhoea is important for control and preventive measures, especially in the African region where there is a high burden of diarrhoeal disease. The present study assessed the prevalence, molecular characteristics, seasonality and associated clinical features of HAdV infection Tanzanian children below two years of age with and without diarrhoea between 2010–2011.

**Methods:**

Stool specimens, demographic and clinical information were collected in 690 cases and 545 controls. All stool samples were screened for HAdV-antigen using ELISA. Positive samples subsequently underwent real-time PCR and sequencing for molecular typing.

**Results:**

HAdV was detected in 37 children, corresponding to a prevalence of 3.5% (24/690) in diarrhoeic and 2.4% (13/545) in non-diarrhoeic children (*P* > 0.05). Among HAdV-infected children, the median age was significantly lower in diarrhoeic than in non-diarrhoeic children (10 vs. 14 months, *P*˂0.001). More than half of HAdV infected (54.2%) were dehydrated as compared to diarrhoeic children without HAdV (45.8%, *P* = 0.01). The proportion of the enteric HAdV type 40/41 in diarrhoeic and non-diarrhoeic children was (50.0%, 12/24) and (46.2%, 6/13) respectively. Other HAdV types detected were; 1, 2, 7, 18, 19 and 31. The prevalence of adenovirus was not significantly different between rainy and dry seasons. HAdV was not detected in the 33 known HIV positive children. There was no significant association between HAdV infection and gender, nutritional status of the child and parent educational level.

**Conclusion:**

The present study provides further evidence of the contribution of adenovirus in causing gastroenteritis in young children, with symptomatic infection being significantly more prevalent in children below one year. We found similar prevalence of adenovirus in non-diarrhoeic children and in diarrhoeic children. This first report on molecular epidemiology of human adenovirus in Tanzania observed diversity of HAdV types that circulate the study setting. The study findings suggest that HAdV is not an important cause of diarrhoea in young HIV-positive children.

**Electronic supplementary material:**

The online version of this article (doi:10.1186/s12879-014-0666-1) contains supplementary material, which is available to authorized users.

## Background

Globally, diarrhoea is ranking as the second cause of death in children [1]. It is estimated that more than one billion diarrhoea episodes occur every year causing up to 700,000 deaths among children younger than 5 years of age. 72% of these deaths occur in children below two years [2]. Enteric viruses have been recognized as a major cause of childhood diarrhoea [3]-[5].

Human adenovirus (HAdV) causes acute diarrhoea sporadically [4],[6],[7], as well as in outbreaks [5]. Besides acute diarrhoea, adenoviruses cause other diseases such as respiratory diseases, conjunctivitis and haemorrhagic cystitis [8]. Adenoviruses have also been associated with persistent infections in both immunocompetent and immunocompromised individuals [8].

Adenoviruses belong to the family *Adenoviridae* and genus *Mastadenovirus*. To date there are over 60 types of adenoviruses identified [9], grouped into seven species A to G on the basis of their resistance to neutralisation by antisera to other known human adenoviruses [10],[11] or genome analysis [11]. The disease pattern of adenoviruses varies according to species. Adenovirus species F, types 40 and 41, has been found to be regularly associated with gastroenteritis and they are referred as enteric adenoviruses. These two types are responsible for 1-20% cases of diarrhoea, especially in young children, both in developed and developing countries [12]-[14]. Other species such as A (types 12, 18 and 31) [15], C (types 1, 2 and 5) and D (types 28, 29, 30, 32, 37, 43 to 46) [8],[16],[17] have also been associated with diarrhoea. Adenoviruses are also increasingly recognized as a cause of infections in immunocompromised hosts, including HIV [18]-[20]. These infections have the potential to cause fatal disseminated disease [21],[22].

Accurate understanding of the relative prevalence of the adenovirus in diarrhoea is important for control and preventive measures. This is especially important in the African region where there is a high burden of diarrhoeal disease and a large reported variation in the reported prevalences for childhood diarrhoea attributable to HAdV ranging from as low as 3.1% in Tunisia [23] to as high as 10.4% in Egypt [24], 19.8% in Ghana [25], 23% in northwest Nigeria [26], and 37.4% in Kenya [20].

In Tanzania, limited data are available regarding the contribution of adenovirus in diarrhoeic children. There are two previous reports that evaluated adenovirus, including type 40/41 only in the study setting [27],[28]. Furthermore, there is a gap of studies on the molecular epidemiology of adenovirus. There are also no studies which have looked at the association between HIV infection and adenovirus. The present study aimed at determining the prevalence of adenovirus in diarrhoeic and non-diarrhoeic children. The study also performed molecular characterisation of the detected adenovirus. Additionally the study evaluated the association between adenovirus with clinical characteristics, HIV status and seasonality.

## Methods

### Study population

This case control study was conducted between August 2010 and July 2011. Details of the study population and data collection have been previously described [29]. Briefly, sample collection was performed during two seasons, starting in August 2010 and in March 2011, aiming for minimum 300 diarrhoeic children and 300 non-diarrhoeic children in each period. The target for diarrhoeic was reached in January 2011 and in June 2011, while enrolment of non-diarrhoeic children continued in February 2011 and July 2011, thus enrolment continued for one complete year. A total of 1266 samples were collected and of these 1235 samples were adequate for detection of adenovirus using ELISA (690 diarrhoeic and 545 non-diarrhoeic children).

### Recruitment of diarrhoeic children

Diarrhoeic children were children hospitalised due to diarrhoea (n = 690) at three major hospitals of Dar es Salaam; Muhimbili National Hospital (MNH), Amana and Temeke Municipal hospitals.

### Recruitment of non-diarrhoeic children

Non-diarrhoeic children (n = 545) were enrolled during the recruitment of diarrhoeic children. These were either children outpatient children attending child health clinics for immunisation and growth monitoring (n = 310) or children admitted to hospital due to diseases other than diarrhoea (n = 235).

### Inclusion and exclusion criteria

Diarrhoeic children included in the study were hospitalised in the diarrhoea wards, with acute or persistent diarrhoea. Diarrhoea was defined as three or more watery stools within 24 hours. An episode of diarrhoea was considered over when two consecutive days pass without diarrhoea. An episode of acute diarrhoea was defined as diarrhoea with duration between 24 hours and less than 14 days. Persistent diarrhoea was defined as diarrhoea for 14 days or more. Non diarrhoeic children included in the study were children without history of diarrhoea for one month prior to enrolment. Children above 24 months of age and cases that could not provide stool sample on the day of admission were excluded from the study. Furthermore diarrhoeic and non-diarrhoeic children whose parent or guardian did not consent to participate in the study were excluded.

### Data collection

Two standardized questionnaires for diarrhoeic and non-diarrhoeic children were used to collect demographic and clinical information, including age (date of birth), sex, place of residence, parent/guardian level of education and history of antibiotic use prior to admission. Consistency of stool and duration of diarrhoea was also recorded. The child’s length and weight measurements were recorded. For diarrhoeic children, additional clinical information was obtained from patient files. This was information on hydration status which was assessed on the day of admission by the attending clinician and HIV testing results. Weight and height measurement was done as previously described [28]. A single stool specimen was collected on inclusion from each child using wide mouthed sterile plastic containers.

### Adenovirus detection

Adenovirus antigen was detected using the commercially available ELISA Kit, ProSpecT™Adenovirus kit designed to detect 51 HAdV types (Oxoid, Hants, UK), with 10% faecal suspensions according to the manufacturer’s instructions.

### Real time PCR for adenovirus genotyping

All HAdV positive samples from ELISA screening underwent real-time PCR for genotyping. DNA extraction has been described before by Moyo *et al*. [29]. DNA was extracted using Magna Pure LC (Roche Diagnostics). Stool samples (50 mg) were mixed 1:10 with Bacterial Lysis buffer (Roche Diagnostics, Mannheim, Germany), and centrifuged at 13.000g for 3 minutes. DNA was extracted from 200μl supernatant using the Magna Pure LC High Performance Total Nucleic Acid Isolation Kit (Roche Applied Science, Mannheim, Germany). Total nucleic acid was eluted and stored at −70°C until analysis.

The primers used for real-time PCR were **Ad F**: 5′-GAC GCY TCG GAG TAC CTG AG-3′ (nt10-29, AF161559) and **Ad R**: 5′-CCY ACR GCC AGI GTR WAI CGM RCY TTG TA-3′ (nt 221–193, AF161559). The TaqMan probe used was **Ad TM**: 5′-CTG GTG CAG TTY GCC CGC-3′ (nt 37–54, AF161559) with FAM labeled as a fluorescent dye on the 5′ end and TAMRA as a fluorescence quencher dye labeled to the 3′ end (TIB Molbiol GmbH, Berlin). In some cases when a larger PCR-product (322 bp) was needed for exact genotyping; an alternative reverse primer (5′-AGCSAGIGARTTGTAAGC-3′) was used. Primers and TaqMan probes were chosen within the conserved region of the hexon-coding gene of adenoviruses [30].

Real time PCR was carried out in 15μL final reaction volume containing; five μL of the DNA-template which was transferred to PerfeCTa Multiplex qPCR SuperMix with uracil-N-glycosidase [12] (Quanta Biosciences), Adeno forward primer (300nM final cons.), Adeno reverse primer (300nm final cons), Adeno TaqMan probe (200nM final cons.) and PCR-grade water. All amplifications were performed using a CFX96 real-time PCR System (Bio-Rad Laboratories, Inc). Amplification conditions were 1 cycle at 45°C for 5 minutes (UNG activation), 1 cycle at 95°C for 3 minutes (polymerase activation), followed by 40 cycles at 95°C for 10 seconds (denaturation) , 55°C for 10 seconds (annealing) and 72°C for 10 seconds (extension). Fluorescence emission was set to be measured at the end of extension step. Positive and negative controls were also included. The described real-time-PCR is able to detect adenovirus from type 1 to type 51. The detection limit of the PCR is approximately 300 copies/ml.

### Sequencing of PCR amplicons

The PCR-product was purified and sequenced using the forward Ad F-primer. Sequencing was performed on an ABI 3130xl DNA Sequencer (Applied Biosystems, Foster city, CA, USA). Nucleotide sequences were analysed using BLAST service at NCBI. The results were compared to known adenovirus sequences in the GenBank by pairwise comparison from multiple alignments using the Genius software package (Biomatters). The phylogenetic tree was constructed using UPGMA and Kimura two-parameter methods [31]. Twenty six sequences with more than 200bp length obtained in this study have been submitted to GenBank with accession numbers: KM000013-KM000038.

### Data analysis

Data was analysed using Statistical Package for the Social Sciences (SPSS for IBM-PC, release 18.0; SPSS Inc., Chicago, IL, US). The prevalence and the median age of adenovirus infection in diarrhoeic and non-diarrhoeic children were compared using chi-square (χ^2^) test and Mann–Whitney U test respectively. To test for significant associations between adenovirus positivity and season, demographic or clinical characteristics, a univariate analysis was performed Weight-for age, and weight for length Z- scores were calculated using EPI Info (USD, Inc., Stone Mountain, GA). A cut off *P*-value of < 0.05 was considered significant.

### Ethical considerations

The study received ethical approval from the Senate Research and Publications Committee of Muhimbili University of Health and Allied Sciences (MUHAS) and from the Regional Committee for Medical and Health Research Ethics (REK) in Western Norway. Permission was obtained from the respective hospital authorities where recruitment of study participants took place (i.e. MNH, Amana and Temeke Hospitals). Written informed consent was also obtained from parent/guardian of the child.

## Results

### Prevalence and distribution of adenovirus by age

Human adenovirus was detected in 37 out of 1235 children. The prevalence of HAdV in diarrhoeic and non-diarrhoeic children did not differ significantly, (3.5%, 24/690 vs. 2.4%, 13/545, *P* = 0.26). There was no significant difference in prevalences of HAdV in non-diarrhoeic children attending child health clinics (3.2%, 10/310) and those admitted to hospital due to diseases other than diarrhoea (1.3%, 3/235, *P* = 0.14).

In both diarrhoeic children and non-diarrhoeic children, the median age was significantly higher in HAdV infected than HAdV non-infected children (10 vs 9 months, *P* = 0.032 and 14.1 vs. 10.9 months, *P* = 0.04). As shown in Table 1, the highest proportion of HAdV infection was in the age group of 7–12 months in diarrhoeic children, P = 0.04. In the age group of zero to six months, only one HAdV was detected in diarrhoeic children while no HAdV was detected in non-diarrhoeic children.

**Table 1:** **Association between demographic**/**clinical characteristics and HAdV infection in diarrhoeic and non**- **diarrhoeic children**

	Diarrhoeic children			Non-diarrhoeic children		
Demographic/ clinical characteristic	HAdV positive	HAdV negative	*P* ^a^	HAdV positive	HAdV negative	*P* ^a^
				N = 13	N = 532	
	N = 24	N = 666				
**Sex**						
Male	15 (62.5)	407 (61.1)		10 (76.9)	286 (53.8)	
Female	9 (37.5)	259 (38.9)	0.89	3 (23.1)	246 (46.2)	0.98
**Age groups in months**						
0-6	1 (4.2)	190 (28.5)		0 (0.0)	120 (22.6)	
7-12	15 (62.5)	328 (49.2)		4 (30.8)	207 (38.9)	
13-18	6 (25.0)	86 (12.9)	0.04	7 (53.8)	152 (28.6)	0.09
19-24	2 (8.3)	62 (9.3)		2 (15.4)	53 (10.0)	
**Parent level of education**						
Primary education	18 (75.0)	513 (77.0)		11 (84.6)	402 (75.6)	
Secondary education	4 (16.7)	133 (20.0)	0.33	2 (15.4)	125 (23.5)	0.73
Higher education	2 (8.3)	20 (3.0)		0 (0.0)	5 (0.9)	
**Type of diarrhoea**						
Acute diarrhoea	22 (91.7)	589 (88.4)	0.66	NA	NA	NA
Persistent diarrhoea	2 (8.3)	77 (11.6)		NA	NA	
**Hydration status**						
Dehydration	13 (54.2)	508 (76.3)	0.01	NA	NA	NA
No dehydration	11 (45.8)	158 (23.7)		NA	NA	
**Nutrition status**						
***i*** ***)*** ***Underweight*** ***(WAZ)***						
Malnourished	15 (62.5)	377 (56.6)	0.57	6 (46.2)	211 (39.7)	0.64
Normal	9 (37.5)	289 (43.4)		7 (53.8)	321 (60.3)	
***ii)*** ***Stunting*** ***(HAZ)***						
Malnourished	18 (75.0)	451 (67.7)	0.46	6 (46.2)	295 (55.5)	0.51
Normal	6 (25.0)	215 (32.3)		7 (53.8)	237 (44.5)	

^a^ *P*-values resulting from univariate analysis.

*NA*: Not Applicable.

### Association between demographic/clinical characteristics and adenovirus infection in diarrhoeic and non-diarrhoeic children

The HIV status was known for 421 children. Twenty six diarrhoeic children and seven non-diarrhoeic children tested positive for HIV. HAdV was not detected in any of these 33 children who tested positive for HIV. HAdV was detected in 2.6% (2/78) HIV negative diarrhoeic children and 3.2% (10/310) HIV negative non-diarrhoeic children.

Table 1 shows that more than half of the adenovirus- infected children with diarrhoea were dehydrated (*P* = 0.013) and that most of them presented with acute symptoms. There was no significant association between HAdV infection and sex of the child or parent/guardian education level and nutritional status of the child (Table 1).

### Seasonality of adenovirus infection

**Figure 1:**
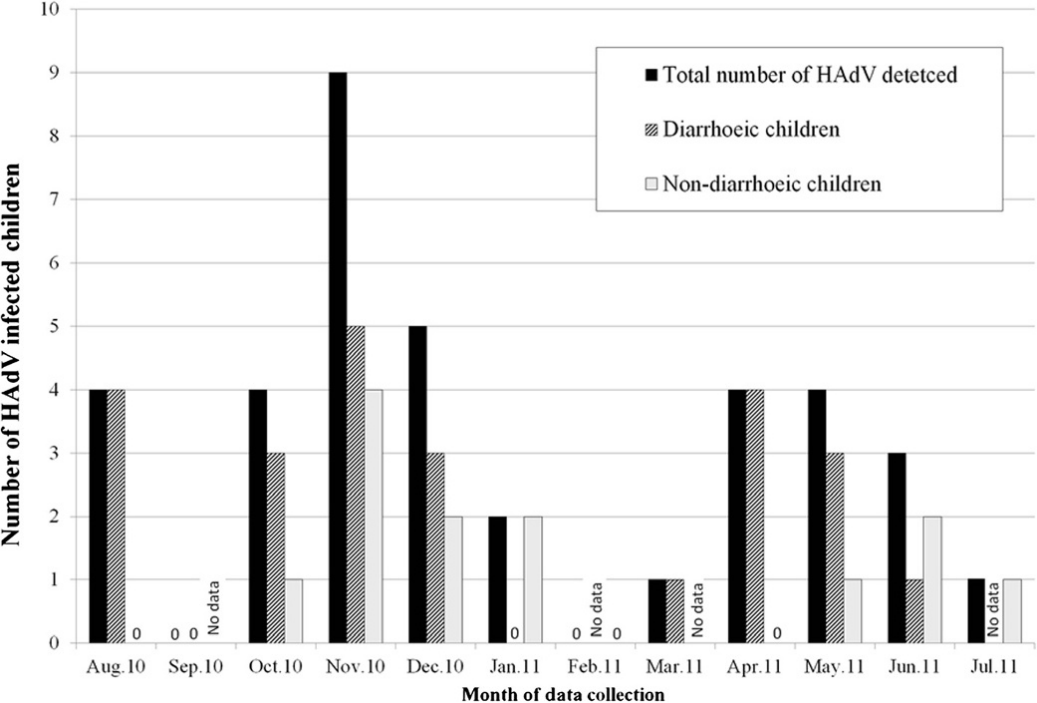
**Seasonal pattern of HAdV detected in diarrhoeic and non**- **diarrhoeic children.** The graph shows total number of HAdV detected (N = 37) and number of HAdV in diarrhoeic and non-diarrhoeic children in each month of the study.

We analysed data on a monthly basis in order to determine the seasonal distribution of HAdV infection (Figure 1). HAdV was not detected in the month of September 2010 and January 2011 in diarrhoeic children. In non-diarrhoeic children, HAdV was not detected in August 2010, February, March and April 2011.

We divided the months of the study according to the season of the year i.e. short rains season, October through December; long rains season, March through May; and the rest were dry months of the year [32]. The prevalence of HAdV was significantly higher during the short rainy season compared to other months (4.5%, 18/396 vs. 2.3%, 19/839, *P* = 0.028, OR 2.0 (95% CI: 1.06 to 3.78). However, when data was combined for both rainy seasons, there was no significant difference between rainy and dry seasons (27/799, 3.4% vs. 10/436, 2.3%, *P* = 0.29). The different types of adenovirus did not show pattern of seasonality.

### Molecular epidemiology

**Figure 2:**
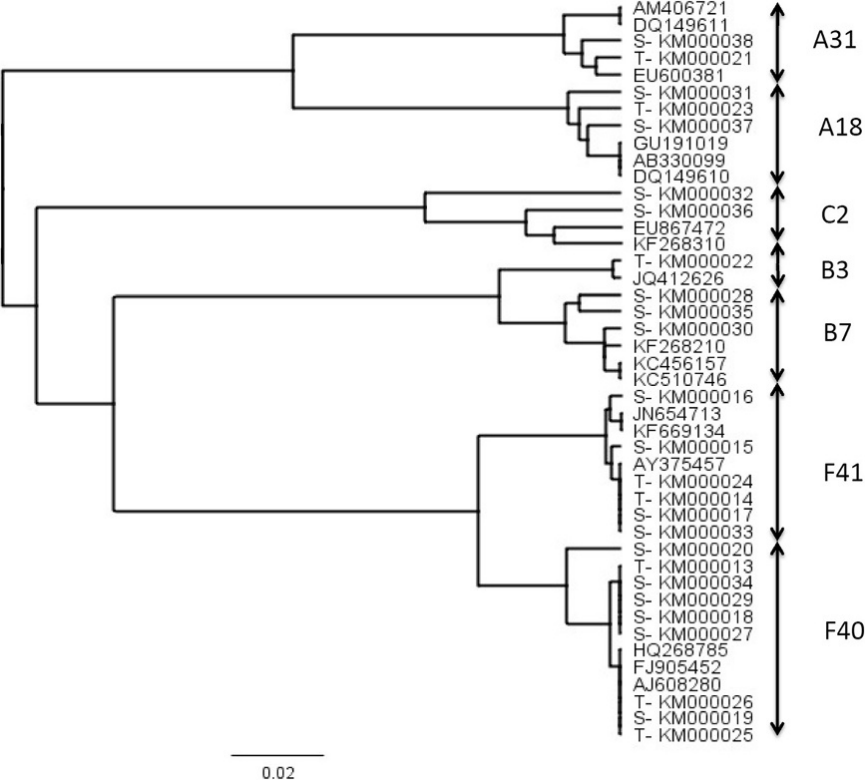
**Phylogenetic tree of HAdV in Dar es Salaam**, **Tanzania during 2010**–**2011.** Phylogenetic tree based on nucleotide sequences of adenovirus hexon gene obtained in this study. Both the study strains and reference strains are indicated by GenBank accession numbers. The accession numbers of the study strains are preceded with S and T (diarrhoeic and non- diarrhoeic specimens respectively). Genius software package was used to build the tree with UPGMA method and bootstrapped with 1000 repetitions. The Kimura-2 substitution model was used. The bar indicates nucleotide substitutions per site.

**Figure 3:**
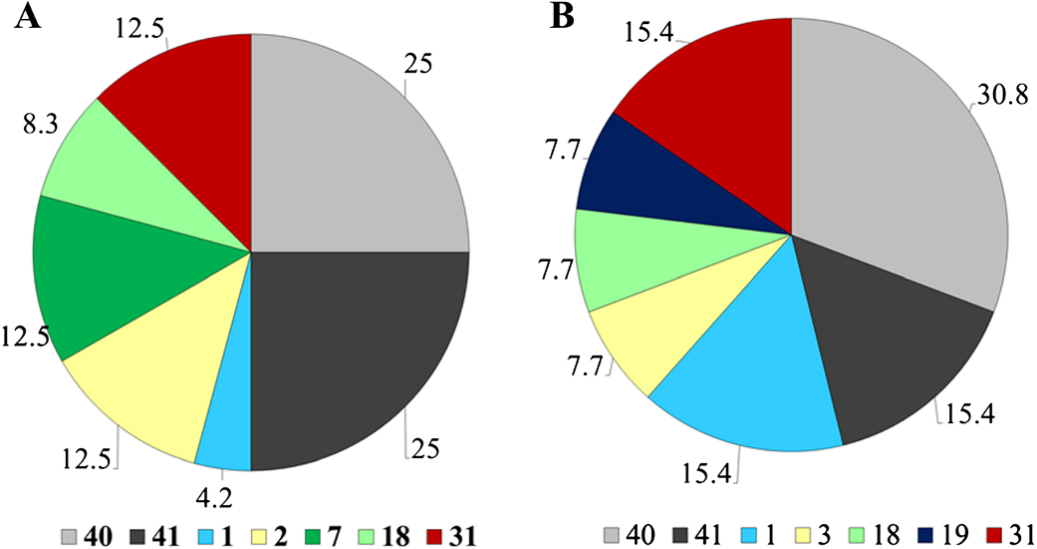
**Distribution HAdV types among adenovirus infected diarrhoeic** **(N** **=** **24)** **and non**-**diarrhoeic** **(N** **=** **13)** **children.** The figure shows distribution of adenovirus types in diarrhoeic **(A)** and non-diarrhoeic children **(B)**. Percentages of each HAdV type are shown on each pie. Coloured pies represent non-enteric HAdV types while a dark grey and a light grey pie represent enteric HAdV types.

The nucleotide sequences of the 37 adenoviruses detected were compared to reference adenovirus strains available in the GenBank by BLAST. A phylogenetic tree was also constructed (Figure 2) from the 26. HAdV nucleotide sequences obtained in this study which were submitted to GenBank (18 diarrhoeic and 8 non-diarrhoeic children). This study found different species and types of HAdV. Among the 24 HAdV detected in diarrhoeic children, seven types were defined, and out of these, 50% (12/24) were enteric adenovirus types 40 and 41 which occurred with equal prevalence (Figure 2). Seven different types were also found among the 13 HAdV in non-diarrhoeic children. Of these 46.2% (6/13) were enteric adenovirus types, and type 40 was more prevalent than type 41 (30.8% vs. 15.4%), as shown in Figure 3. The proportions of enteric adenoviruses (type 40 and 41) were not significantly different in diarrhoeic and non-diarrhoeic children (50%, 12/24 vs. 46%, 6/13, *P* = 0.82). Similarly the proportion of non-enteric adenovirus did not differ between diarrhoeic and non-diarrhoeic children (50%, 12/24 vs. 53.85%, 7/13, *P* = 0.82).

## Discussion

The molecular epidemiology of human adenovirus species and types from Tanzania is described for the first time in the present study. The study also provides the prevalence of human adenoviruses in hospitalised diarrhoeic children and non-diarrhoeic children.

The prevalence of enteric adenovirus in children in the current study (1.8%) was not significantly different from what was reported by our group previously (2.6%), *P* = 0.64 [28]. The slightly higher total adenovirus prevalence observed in the current study could be due to the use of an Enzyme Immune Assay (EIA) able to detect 51 types of adenovirus compared to the EIA method specific to enteric adenovirus 40/41 which was used in the previous study [28]. Despite the fact that we found higher prevalence of adenovirus in the present study compared to the previous study [28], the EIA method used for detection of adenovirus in the current study has a limitation, it is reported to be less sensitive when compared with PCR [33]-[35]. However, PCR is relatively expensive compared to ELISA especially when the expected prevalence is low. Different methods have been employed for detection of adenovirus in studies conducted in African countries. Studies which detected HAdV using PCRhave generally reported higher prevalences [20],[26],[36] compared to studies which employed EIA [23],[37].

The finding of an almost similar prevalence of HAdV in diarrhoeic and non-diarrhoeic children, but at higher median age of infection in non-diarrhoeic children, suggest prolonged shedding of adenovirus in stool after previous infection of more than one month prior to the study. Alternatively, it could be asymptomatic adenovirus infections in children who may have acquired immunity from previous infections.

We found that the majority of HAdV infected diarrhoeic children were dehydrated. This concurs with reports from other developing countries [38] re-affirming that adenovirus cause severe diarrhoea.

Sequence analysis showed that a wide variety of HAdV species (five) and types (nine) circulate among diarrhoeic and non-diarrhoeic children in Dar es Salaam. The enteric adenoviruses (type 40 and 41) were found to constitute approximately half the HAdV positive cases, both in the diarrhoeic and non-diarrhoeic groups of children. As have been reported in other studies [39],[40]. Adenovirus type 40 and 41 occurred at equal frequency in diarrhoeic children. In the group of non-diarrhoeic children, adenovirus type 40 was more prevalent than type 41. Some studies have reported antigenic drift of adenovirus type 41, leading to an increase of adenovirus type 41 at the expense of adenovirus type 40 [12],[13]. In order to detect antigenic drift in the study setting, future studies are needed over a long period of time, as reported elsewhere [12],[13],[41].

Human adenovirus types 1, 2, 3, and 7 which are associated with respiratory infections [8],[42], are also shown to be associated with diarrhoeal disease [16]. In the present study these adenovirus types were detected from both diarrhoeic and non-diarrhoeic children. When these HAdV types infect respiratory sites, they can be shed in the faeces of an infected person for months [8],[42]. Hence findings of this study could support the theory of prolonged shedding of these human adenovirus species in faeces. However, we cannot rule out their role in diarrhoea aetiology because these types have also been reported to cause diarrhoea [16].

Despite the fact that diarrhoea is known as a major source of morbidity and mortality in HIV infected children, particularly in developing countries [43]-[45], the causes of diarrhoea in HIV-infected children are not well understood. There is one published report on the role of adenovirus in diarrhoea from HIV positive children in Africa [20]. In this study conducted in Kenya adenovirus was not detected among children aged below two years who were known HIV positive [20]. Likewise, in the present study adenovirus was not detected in HIV infected children. These two African studies and other studies [46] suggest that adenovirus is not an important cause of diarrhoea in young HIV positive children.

In the present study we observed higher prevalence of HAdV during the short rain months of the study period but not overall for the two rainy seasons combined. In a study conducted in Bangladesh a high prevalence of adenovirus infection during the rainy season was reported [38]. However, other studies have reported no specific seasonal pattern of adenovirus infection [41],[47].

## Conclusion

The present study provides further evidence of the contribution of adenovirus in causing gastroenteritis in young children, with infection being significantly more prevalent in children below one year. We found similar prevalence of adenovirus in non-diarrhoeic children as in diarrhoeic children. This first report on molecular epidemiology of human adenovirus in Tanzania, we found an equal proportion of enteric types and non-enteric types in both diarrhoeic and non-diarrhoeic children.

## Authors’ original submitted files for images

Below are the links to the authors’ original submitted files for images. Authors’ original file for figure 1 Authors’ original file for figure 2 Authors’ original file for figure 3
