# Supplementary figures and images for: Prevalence and molecular characterisation of human adenovirus in diarrhoeic children in Tanzania; a case control study
Source: BMC Infect Dis. 2014 Dec 12;14:666. doi: 10.1186/s12879-014-0666-1 (PMC4266963; doi:10.1186/s12879-014-0666-1)

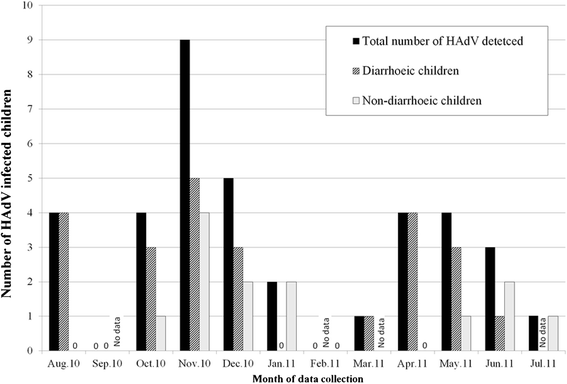

Supplement: Supplementary file 1 — Authors’ original file for figure 1 [file 12879_2014_666_MOESM1_ESM.gif]

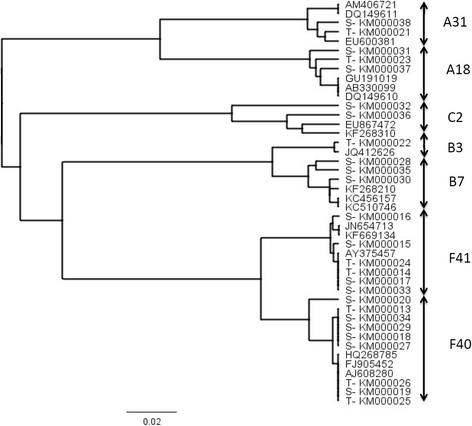

Supplement: Supplementary file 2 — Authors’ original file for figure 2 [file 12879_2014_666_MOESM2_ESM.gif]

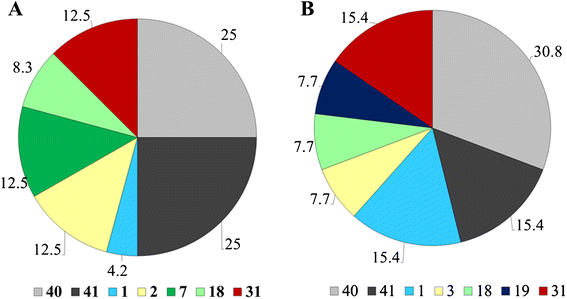

Supplement: Supplementary file 3 — Authors’ original file for figure 3 [file 12879_2014_666_MOESM3_ESM.gif]
